# Prognostic value of the neutrophil-to-lymphocyte ratio and platelet-to-lymphocyte ratio in laryngeal cancer: What should we expect from a meta-analysis?

**DOI:** 10.3389/fonc.2022.945820

**Published:** 2022-08-10

**Authors:** Xianyang Hu, Tengfei Tian, Qin Sun, Wenxiu Jiang

**Affiliations:** ^1^ School of Basic Medical Sciences, Shanghai Medical College of Fudan University, Shanghai, China; ^2^ Department of Otolaryngology-Head and Neck Surgery, Eye Ear Nose and Throat Hospital, Fudan University, Shanghai, China

**Keywords:** prognosis, meta-analysis, neutrophil-to-lymphocyte ratio, platelet-to-lymphocyte ratio, laryngeal cancer

## Abstract

**Background:**

Although many studies have shown the predictive value of the high neutrophil-to-lymphocyte ratio (NLR) and platelet-to-lymphocyte ratio (PLR) for various cancers, there are conflicting reports regarding their role in laryngeal cancer. This study aimed to evaluate the relationship between high NLR/PLR and laryngeal cancer prognosis with the help of meta-analysis.

**Methods:**

PubMed, Embase and other databases were used to search relevant studies. The pooled hazard ratio (HR) and 95% confidence interval (CI) were calculated using either the random-effect-model or fixed-effect model. Sensitivity analyses and subgroups were used to explore potential sources of heterogeneity. Publication bias was also adopted.

**Result:**

5716 patients from 20 studies were involved in this meta-analysis. Pooled observed survival (OS) (HR=1.70, 95%CI, 1.41-2.04, p<0.001), progression-free survival (PFS) (HR=1.81, 95%CI, 1.47-2.23, p<0.001), and disease-free survival (DFS) (HR=1.86, 95%CI, 1.45-2.38, p<0.001) showed the prediction of high NLR for poor prognosis. It also suggested that high PLR predicted poor OS (HR=1.89, 95%CI, 1.21-2.94, p<0.001).

**Conclusion:**

This study indicated that high NLR was associated with poor OS, PFS, and DFS in laryngeal cancer patients, and high PLR was related to poor OS. Both could be potential predictors of prognosis.

## Introduction

Laryngeal cancer is one of the most common malignancies, accounting for about 2.4 percent of new malignancies worldwide (1, 2). In 2015, there were 16,300 new cases of laryngeal cancer and 14,500 deaths in China (3). The American Cancer Society also estimates that 12,470 new cases will be diagnosed in the United States in 2022, and 3,820 people will die of laryngeal cancer (4). Despite the continuous progress of radiation therapy, transoral laser microsurgery and other laryngeal cancer treatments (5), no significant improvement in the observed survival (OS) or relative survival rate (RS) has been reported (6). Currently, clinical treatment is selected mainly according to the TNM Classification by the American Joint Commission on Cancer. However, this system focuses only on the anatomical features of tumor size and metastasis, which is not a reliable prognosis for the disease. Prognosis varies among patients with the same stage (7), while immune status (8) and pathological types (9) contribute to prognosis more. The interaction between psychological and functional changes caused by cancer treatment is also important (10). It is essential to find easily accessible new evaluation markers to assess the survival outcome of laryngeal cancer. It has been shown that inflammatory responses can promote cancer development and affect prognosis (11). Tumor-related inflammatory markers have become a focus of many researchers. Previous studies have identified that lymphocyte-to-monocyte ratio, interleukin-12 receptor, immune-inflammatory index and the interleukin-23 receptor can be prognostic indicators for laryngeal cancer (12, 13). High neutrophil/lymphocyte level and low lymphocyte level are closely related to tumor invasion and progression (14–16). As new inflammatory indicators, Neutrophil-to-lymphocyte ratio (NLR) and platelet-to-lymphocyte ratio (PLR) also have good prognostic value for gastric cancer (17), esophageal cancer (18), bladder cancer (19), breast cancer (20), and ovarian cancer (21). Recent studies showed that high pre-treatment NLR/PLR is a sign of poor prognosis in laryngeal cancer (22–24), but this result has not been confirmed in some other studies (22, 25, 26). Therefore, the role of NLR/PLR in the prognosis of laryngeal cancer remains controversial. Meta-analysis is recognized as a powerful statistical tool that can break down the limitations of different individual studies. It can be used to synthesize different studies and obtain objective and scientific results. In this meta-analysis, we aimed to assess the value of NLR and PLR in the prognosis of laryngeal cancer through meta-analysis.

## Materials and methods

### Search strategy

A systematic search of PubMed, Embase, and other databases was conducted from March 1, 1995, to March 1, 2022. Only English literature could be considered. Both Mesh terms and free-text words were used for article search. The detailed search strategy is as follows: (‘Platelet-to-lymphocyte ratio’ OR ‘platelet-lymphocyte ratio’ OR ‘PLR’ OR ‘platelet to lymphocyte ratio’ OR ‘platelet lymphocyte ratio’ OR ‘neutrophil lymphocyte ratio’ OR ‘neutrophil-to-lymphocyte ratio’ OR ‘neutrophil-lymphocyte ratio’ OR ‘NLR’ OR ‘neutrophil to lymphocyte ratio’ OR ‘neutrophil lymphocyte ratio’) AND (‘Laryngeal Neoplasm’ OR ‘Larynx Neoplasms’ OR ‘Larynx Neoplasm’ OR ‘Cancer of Larynx’ OR ‘Larynx Cancers’ OR ‘Laryngeal Cancer’ OR ‘Laryngeal Cancers’ OR ‘Larynx Cancer’ OR ‘Cancer of the Larynx’ OR ‘Laryngeal Neoplasms’). All reference lists for candidate articles were searched independently by two authors (Xianyang Hu and Tengfei Tian). In case of disagreement, the decision is left to the third author (Qin Sun).

### Inclusion criteria

Eligibility criteria are as follows: (1) Studies explored the relationship between pre-treatment NLR or PLR and the prognosis of patients with laryngeal cancer. (2) PLR and NLR were measured by serum-based methods. (3) Studies used COX proportional risk analysis to calculate hazard ratios (HR) and their 95% confidence intervals (95% CI) for OS, progression-free survival (PFS), or disease-free survival (DFS), and provided specific data. (4) Score ≥6 according to the Newcastle-Ottawa Scale (NOS). Two authors independently determined whether the candidate article was selected (Xianyang Hu and Tengfei Tian), and any disagreement was resolved by the third author (Qin Sun).

### Exclusion criteria

Exclusion criteria are as follows: (1) Abstracts, case reports, letters, reviews, meta-analysis, system reviews, or non-clinical articles. (2) Articles completed in a language other than English. (3) Studies lacked specific data for HR, 95% CI or p values. (4) Studies data is doubtful (The table shows that high PLR is associated with a good prognosis, which is inconsistent with the description of survival curve in this paper). (5) Duplication of data between studies. Two authors independently determined whether the candidate article was dropped (Xianyang Hu and Tengfei Tian), and any disagreement was resolved by the third author

### Data extraction

For all selected articles, we recorded the following information: the author, the year of publication, time of data collection, study region, study design, the number of patients, diagnosis method, the age of patients, tumor type, follow-up time, cut-off value of PLR and NLR, outcomes and HRs with 95%CI.

### Quality assessment

The quality of the included studies was assessed by NOS, which includes three parts: selection, comparability, and outcome assessment. Each of the three parts has a different score (selection: 0-4, comparability: 0-2, outcome assessment: 0-3). Studies with NOS ≥ 6 were considered high quality. Two authors completed this work (Xianyang Hu and Tengfei Tian).

### Statistical analysis

STATA statistical software (version 12.0) was used for all statistical analyses. For the effect of NLR and PLR on prognosis, we used the pooled HR and 95%CI of OS, PFS, and DFS to assess. When studies had both univariate and multivariate analysis results, we preferred multivariate analysis. Higgins I-squared statistic and Cochran’s Q test were used to assess the heterogeneity. I2>56% or a P heterogeneity<0.1 indicated significant heterogeneity. If considerable heterogeneity existed, the random-effect model would be appropriate. Otherwise, the fixed-effect model would be used. Due to the significant heterogeneity, subgroup analysis, and sensitivity analysis were used to find the sources of heterogeneity. Also, meta-regression analysis was applied to verify the reliability of the results. Publication bias was assessed using Begg’s test and Egger’s tests. For all statistically significant analyses, a two-sided p needed to be less than 0.05.

## Results

### Search information

**Figure 1:**
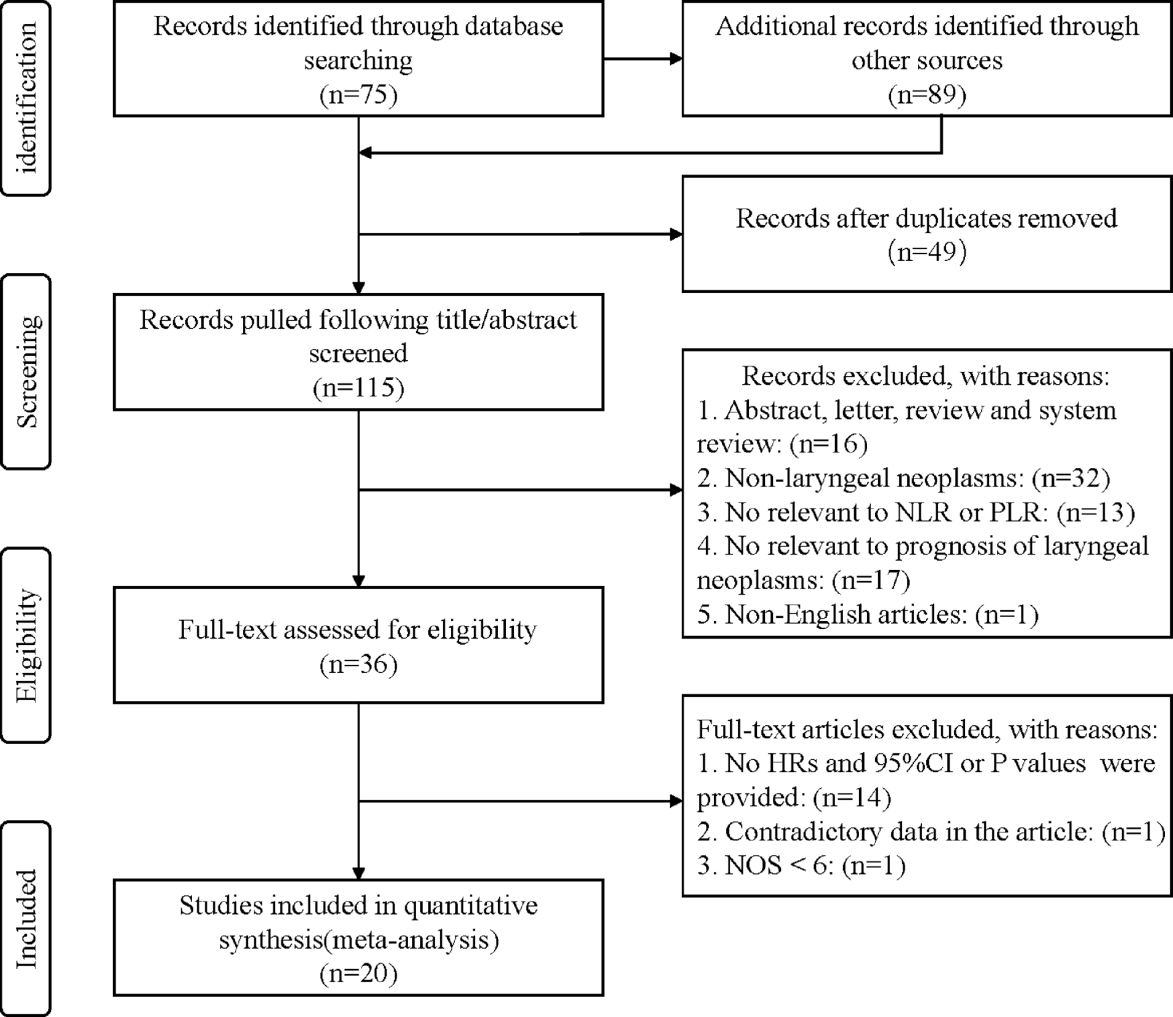
Flow chart of study search and selection.

First, 115 studies were identified through a preliminary data search. Second, by scanning titles and abstracts, sixteen studies were left out as they were non-clinical articles. Forty-five studies were removed because they were not related to the subjects. Seventeen studies were excluded because they were not associated with prognosis. One non-English study was excluded. Afterward, thirty-six articles were eligible for full-text review. Fourteen studies without performing HRs and 95%CI or P values were regarded as lacking data and removed. One article with conflicting data was excluded. Finally, twenty studies with 5716 patients were included. The flow chart shows the detailed selection process (**Figure 1**).

### Characteristics of eligible studies

**Figure 2:**
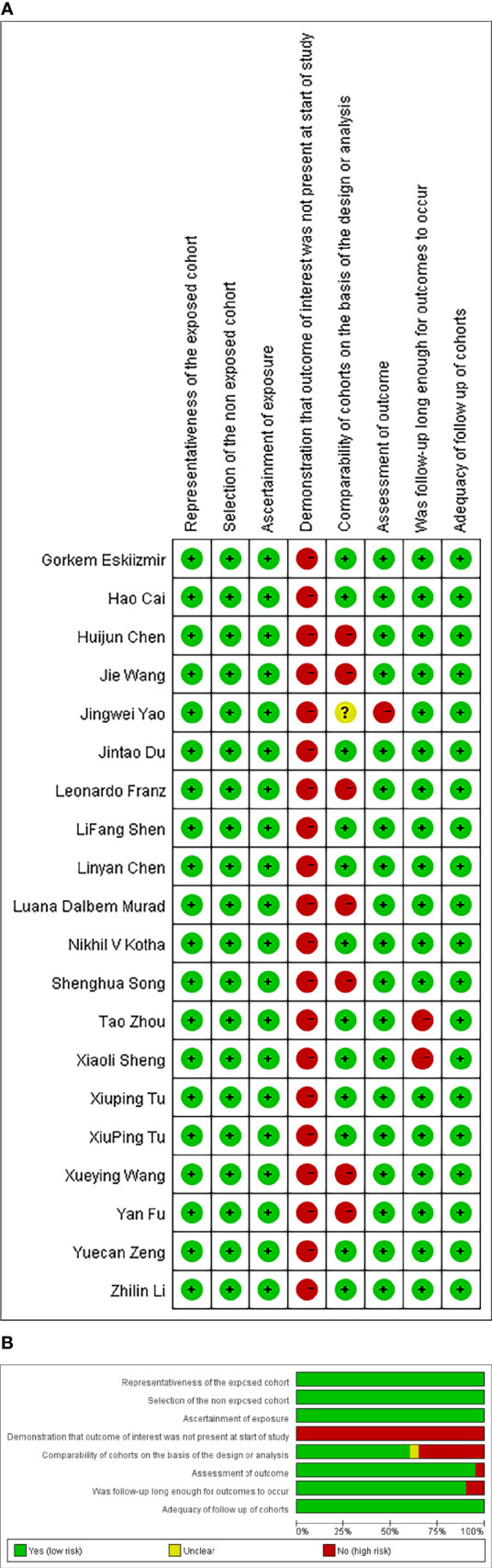
Quality assessment of the included studies.

**Table 1** summarizes the main characteristics of the selected studies. The meta-analysis includes twenty studies published in English from 2015 to 2021. The number of people included in the selected studies ranged from 60 to 1047. All studies were retrospective. These studies were conducted between 1990 and 2018, with sixteen studies from China and four from the United States, United Kingdom, Turkey, and Italy. Eighteen studies focused on Laryngeal Squamous Cell Carcinoma (LSCC), and two studies focused on all types of laryngeal cancers. Fifteen studies used pathology diagnosis, while five used other diagnostic methods or did not mention. All studies recorded pre-treatment NLR or PLR, and some studies reported both pre-and post-treatment data. The post-treatment data were omitted. Fewer than four studies reported the effects of NLR on cancer-specific survival, non-laryngeal cancer survival, recurrence-free survival, and locoregional recurrence-free survival. Therefore, only OS, DFS, and PFS were selected as endpoints for NLR. For the same reason, only OS was selected as the endpoint of PLR. The NOS scores of included studies varied from 6 to 8. **Figure 2** shows the details of NOS scoring.

**Table 1:** Main characteristics of the studies included in this meta-analysis.

No.	Author	Year	During	Study region	Design	Patients(number)	Diagnostic method	Age (years, median and range)	Disease Type	Follow-up (months, median and range)	NLR Cut-off (mg/L)	PLR Cut-off (mg/L)	Outcome	HR Analysis	NOS
1	Jingwei Yao (27)	2021	2012-2017	China	Retrospective	60	pathology	60	M	NA	2.8	–	OS	M	6
2	Xueying Wang (28)	2021	2011-2014	China	Retrospective	412	pathology	NA	LSCC	59.9(1.9~83.2)	NA	NA	OS	M	6
3	LiFang Shen (29)	2021	2009-2018	China	Retrospective	338	pathology	63 (38–86),	M	NA	2.4	122.9	OS	M	8
4	Luana Dalbem Murad (30)	2021	2006-2011	Brazil	Retrospective	168	pathology	61.2 (41–88)	LSCC	60	2.02	–	OS	M	6
5	Zhilin Li (31)	2021	2008-2013	China	Retrospective	147	pathology	60 (37–85)	LSCC	54(3-101)	1.88	117.36	OS/PFS	M	8
6	Nikhil V Kotha (32)	2021	2000-2018	America	Retrospective	1047	others	63(32-90)	LSCC	52	4.17	–	OS/CSS/NCS	M	8
7	Leonardo Franz (33)	2021	NA	Italy	Retrospective	60	pathology	64.5(60-69)	LSCC	58(29-90.5)	2.68	–	DFS	NA	6
8	Hao Cai (22)	2021	2008-2014	China	Retrospective	203	others	62.45(33-85)	LSCC	NA	2.41	110.94	OS/DFS	NLR(OS): M, Others: U	8
9	Huijun Chen (34)	2020	2009-2015	China	Retrospective	473	pathology	63(38-90)	LSCC	NA	1.96	103.956	OS/RFS	U	6
10	Tao Zhou (23)	2019	2008-2013	China	Retrospective	232	others	63 (39-81)	LSCC	27.3(8.7-45.9)	2.38	116	OS/DFS	M	7
11	Shenghua Song (35)	2019	2009-2015	China	Retrospective	137	pathology	62(40-84)	LSCC	43	2.96	109.54	OS/RFS	U	6
12	Xiaoli Sheng (36)	2019	2008-2015	China	Retrospective	110	others	61.5(43-85)	LSCC	NA	2.22	–	OS/DFS	M	7
13	Gorkem Eskiizmir (37)	2019	2002-2015	Turkey	Retrospective	229	others	59(31-88)	LSCC	39.5(1-107)	4	–	OS/DFS/LRFS	M	8
14	XiuPing Tu (24)	2018	2006-2011	China	Retrospective	290	pathology	62(41-86)	LSCC	64(3-102)	2.22	114	OS	NLR:M, PLR: U	8
15	Jintao Du (38)	2018	2008-2013	China	Retrospective	653	pathology	61 (54-67)	LSCC	NA	3.18	–	OS/PFS	M	8
16	Linyan Chen (39)	2018	2010-2014	China	Retrospective	361	pathology	60(35-87)	LSCC	47(4-98)	2.45	114	OS/PFS	NLR: M, PLR: U	8
17	Yuecan Zeng (40)	2016	2007-2013	China	Retrospective	115	pathology	58(45-75)	LSCC	45	3	–	OS/PFS	M	8
18	Jie Wang (16)	2016	2008-2012	China	Retrospective	120	pathology	60.6 (40–81)	LSCC	NA	2.79	112	OS/RFS	NLR: U, PLR: M	6
19	Yan Fu (41)	2016	1990-2010	China	Retrospective	420	pathology	60 (33–84).	LSCC	NA	2.59	–	OS/CSS	M	6
20	Xiuping Tu (42)	2015	2006-2011	China	Retrospective	141	pathology	59(36-87)	LSCC	51(5-102)	2.17	–	OS/DFS	M	8

NLR, neutrophil-to-lymphocyte ratio; PLR, platelet-to-lymphocyte ratio; NA, not available; LCSS, Laryngeal Squamous Cell Carcinoma; others, diagnostic methods are not available or other diagnostic methods; M, multiple types of laryngeal cancer; LSCC, Laryngeal Squamous Cell Carcinoma; OS, overall survival; PFS, progression-free survival; DFS, disease-free survival; RFS, recurrence-free survival; CSS, cancer-specific survival; NCS, non-larynx cancer survival; LRFS, locoregional recurrence-free survival; M, multivariate; U, univariate.

### Impact of NLR on OS

**Figure 3:**
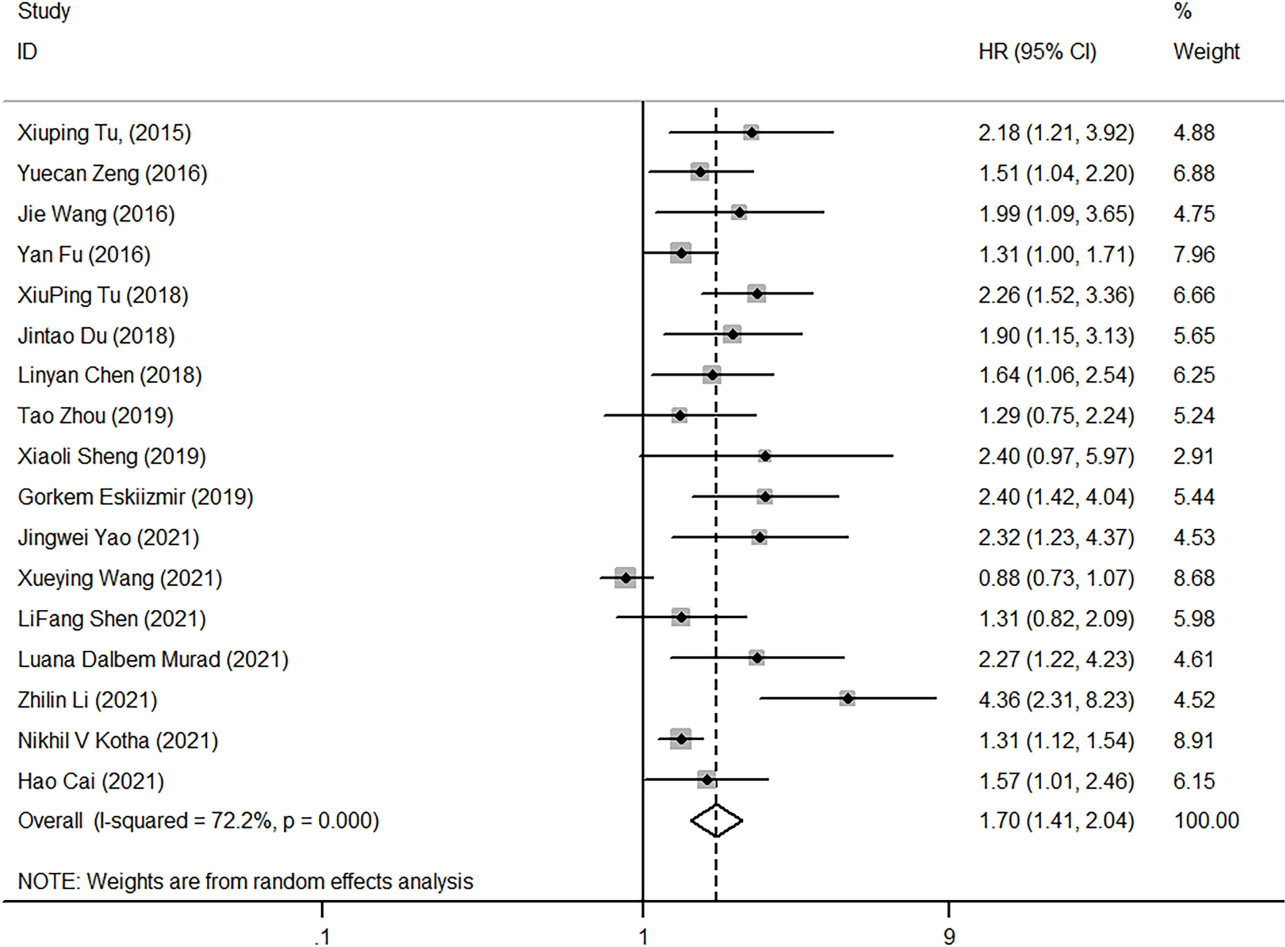
Forest plot of HR for the impact of NLR on OS. NLR, neutrophil-to-lymphocyte ratio; OS, overall survival; HR, hazard ratio; CI, confidence interval.

Seventeen studies explored the correlation between NLR and OS, with heterogeneity testing showing high (I2 = 72.2%, p<0.001). The combined HR (HR=1.70, 95%CI, 1.41-2.04, p<0.001) resulting from the random-effect model showed that high NLR correlates closely with poor OS (**Figure 3**).

Factors that might contribute to heterogeneity, such as diagnostic method, follow-up time, tumor type, NLR cutoff, study area, NOS score, and sample size, were selected. Subgroup analysis was then performed based on these, High NLR had predictive value for the prognosis of laryngeal cancer when NLR cut-off<3 mg/L (HR=1.73, 95%CI, 1.51-1.99), and the same when NLR cut-off≥3 mg/L (HR=1.43, 95%CI, 1.25-1.64). The heterogeneity among subgroups was not significant (cut-off<3, I2 = 43.70, p=0.052; cut-off≥3, I2 = 51.90%, p=0.101), suggesting that NLR cut-off was one of the major reasons for the high heterogeneity. Several studies also confirmed that the NLR with a cut-off of 3 is an important prognostic factor (19). As revealed in **Table 2**, diagnostic method (I2 = 72.20%, p < 0.001), follow-up time (I2 = 82.00%, p<0.001), tumor type (I2 = 72.20%, p<0.001), region of study (I2 = 72.20%, p<0.001), NOS score (I2 = 72.20%, p<0.001), and sample size (I2 = 72.20%, p<0.0010) also worked on the overall heterogeneity.

**Table 2:** Main results of the subgroup analyses for the impact of NLR on OS.

Analysis	Number	Fixed-effects model		Random-effects model		Heterogeneity	
		HR(95%CI)	P	HR(95%)	P	I2	Ph
Diagnostic method	17	1.395(1.280-1.520)	0	1.697(1.409-2.044)	0	72.20%	0.000
pathology	12	1.763(1.358-2.288)	0	1.546(1.212-1.973)	0	78.50%	0.000
others	3	1.386(1.241-1.547)	0	1.409(1.227-1.617)	0	37.60%	0.170
Follow-up	10	1.346(1.219-1.487)	0	1.735(1.322-2.276)	0	82.00%	0.000
<50months	7	1.289(1.127-1.475)	0	1.734(1.158-2.598)	0.008	85.70%	0.000
≥50months	3	1.418(1.223-1.644)	0	1.801(1.135-2.857)	0.012	71.50%	0.030
tumor Type	17	1.395(1.280-1.520)	0	1.697(1.409-2.044)	0	72.20%	0.000
M	2	1.602(1.101-2.330)	0.014	1.672(0.963-2.902)	0.068	50.40%	0.156
LSCC	15	1.384(1.267-1.512)	0	1.704(1.394-2.082)	0	74.60%	0.000
NLR Cut-off	16	1.570(1.426-1.730)	0	1.757(1.503-2.054)	0	49.20%	0.014
<3	12	1.730(1.508-1.985)	0	1.837(1.512-2.231)	0	43.70%	0.052
≥3	4	1.429(1.247-1.637)	0	1.597(1.233-2.069)	0	51.90%	0.101
Study region	17	1.395(1.280-1.520)	0	1.697(1.409-2.044)	0	72.20%	0.000
China	14	1.383(1.244-1.537)	0	1.698(1.350-2.135)	0.012	74.30%	0.000
Non-China	3	1.418(1.223-1.644)	0	1.801(1.135-2.857)	0	71.50%	0.030
NOS	17	1.395(1.280-1.520)	0	1.697(1.409-2.044)	0	72.20%	0.000
6	5	1.138(0.987-1.312)	0.074	1.523(1.025-2.263)	0.037	80.70%	0.000
7	2	1.525(0.955-2.436)	0.077	1.579(0.896- 2.780)	0.114	23.50%	0.253
8	10	1.525(0.955-2.436)	0	1.805(1.465- 2.223)	0	61.00%	0.006
Sample size	17	1.395(1.280-1.520)	0	1.697(1.409- 2.044)	0	72.20%	0.000
<200	7	2.103(1.695-2.609)	0	2.190(1.683-2.850)	0	28.00%	0.214
≥200	10	1.290(1.175-1.417)	0	1.465(1.196-1.796)	0	72.50%	0.000

M, multiple types of laryngeal cancer; LSCC, Laryngeal Squamous Cell Carcinoma; HR, hazard ratio; NLR, neutrophil-to-lymphocyte ratio; CI, confidence interval.

### Impact of NLR on PFS and DFS

**Figure 4:**
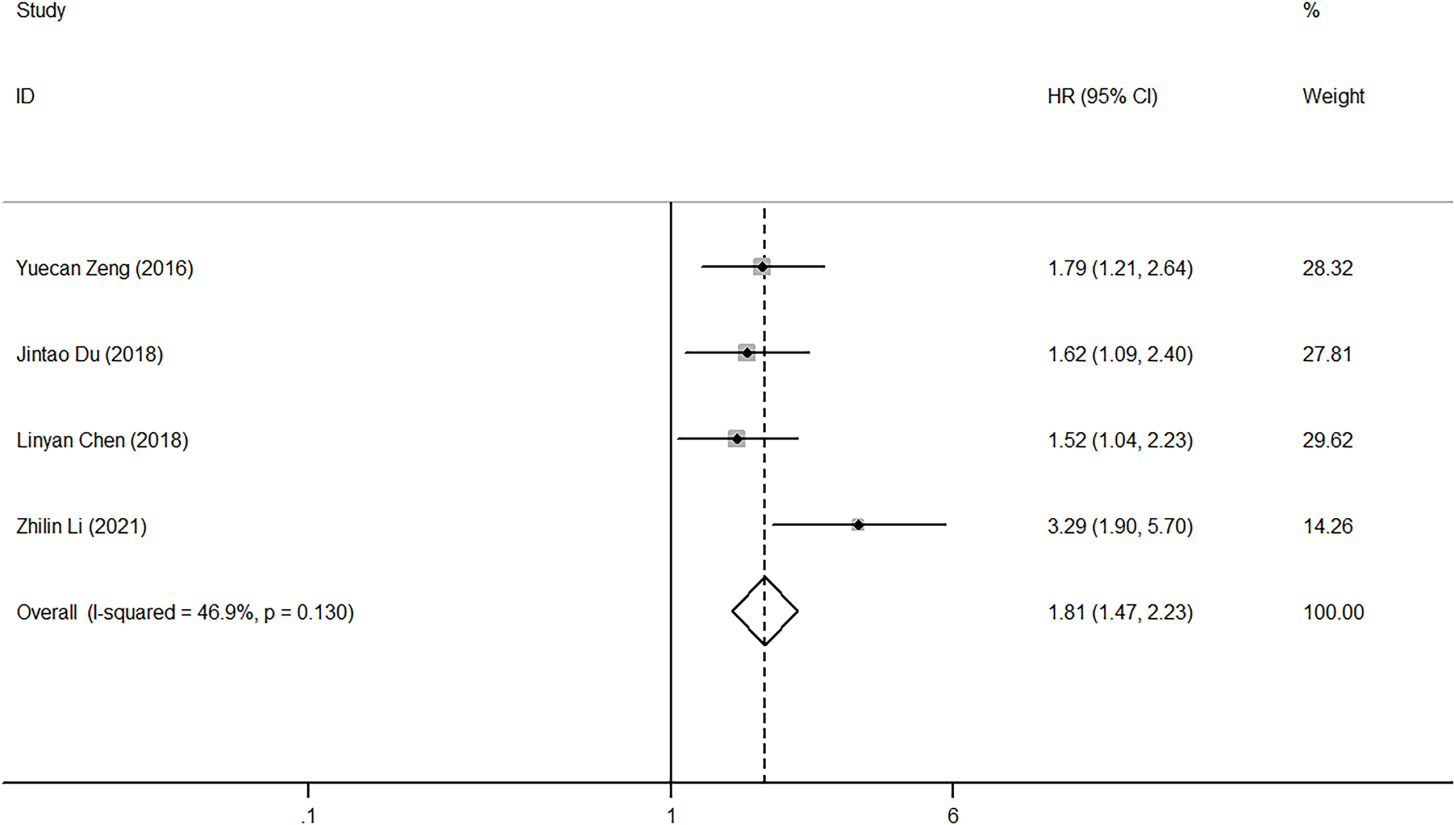
Forest plot of HR for the impact of NLR on PFS. NLR, neutrophil-to-lymphocyte ratio; PFS, progression-free survival; HR, hazard ratio; CI, confidence interval.

**Figure 5:**
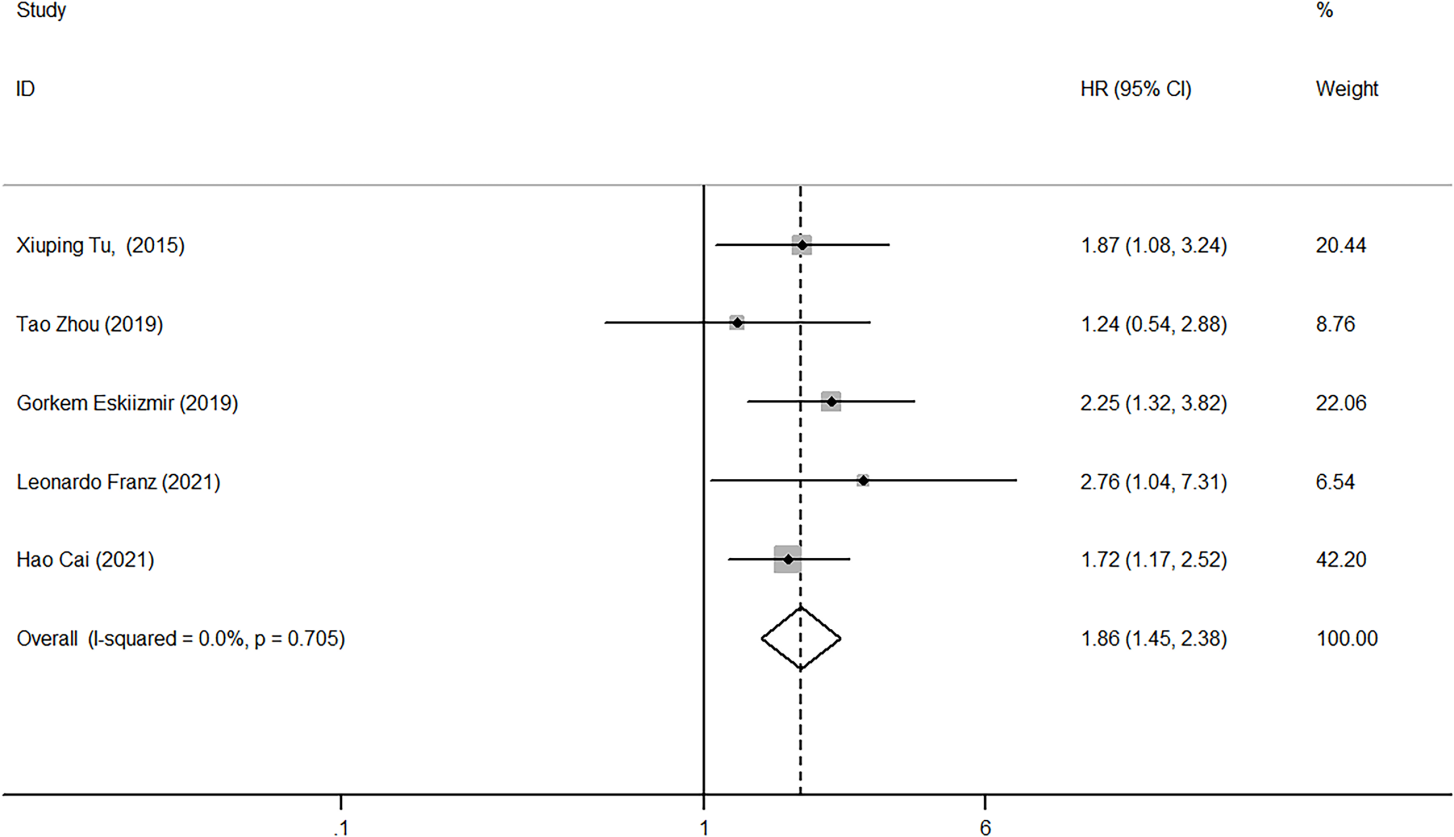
Forest plot of HR for the impact of NLR on DFS. NLR, neutrophil-to-lymphocyte ratio; DFS, disease-free survival; HR, hazard ratio; CI, confidence interval.

Regarding the effect of NLR on PFS, heterogeneity was low among the four studies (I2 = 46.9%, p=0.13). Pooled HR (HR=1.81, 95%CI, 1.47-2.23, p<0.001) calculated by the fixed-effect model showed a correlation between high NLR and poor PFS (**Figure 4**). Five studies reported the impact of NLR on DFS, and their heterogeneity was also mild (I2 = 0, p=0.705), so the fixed-effect model was applied. The result showed that high NLR predicted low DFS in laryngeal cancer (HR=1.86, 95%CI, 1.45-2.38, p<0.001) (**Figure 5**). Because of limited study information, no subgroup analysis was performed.

### Impact of PLR on OS

**Figure 6:**
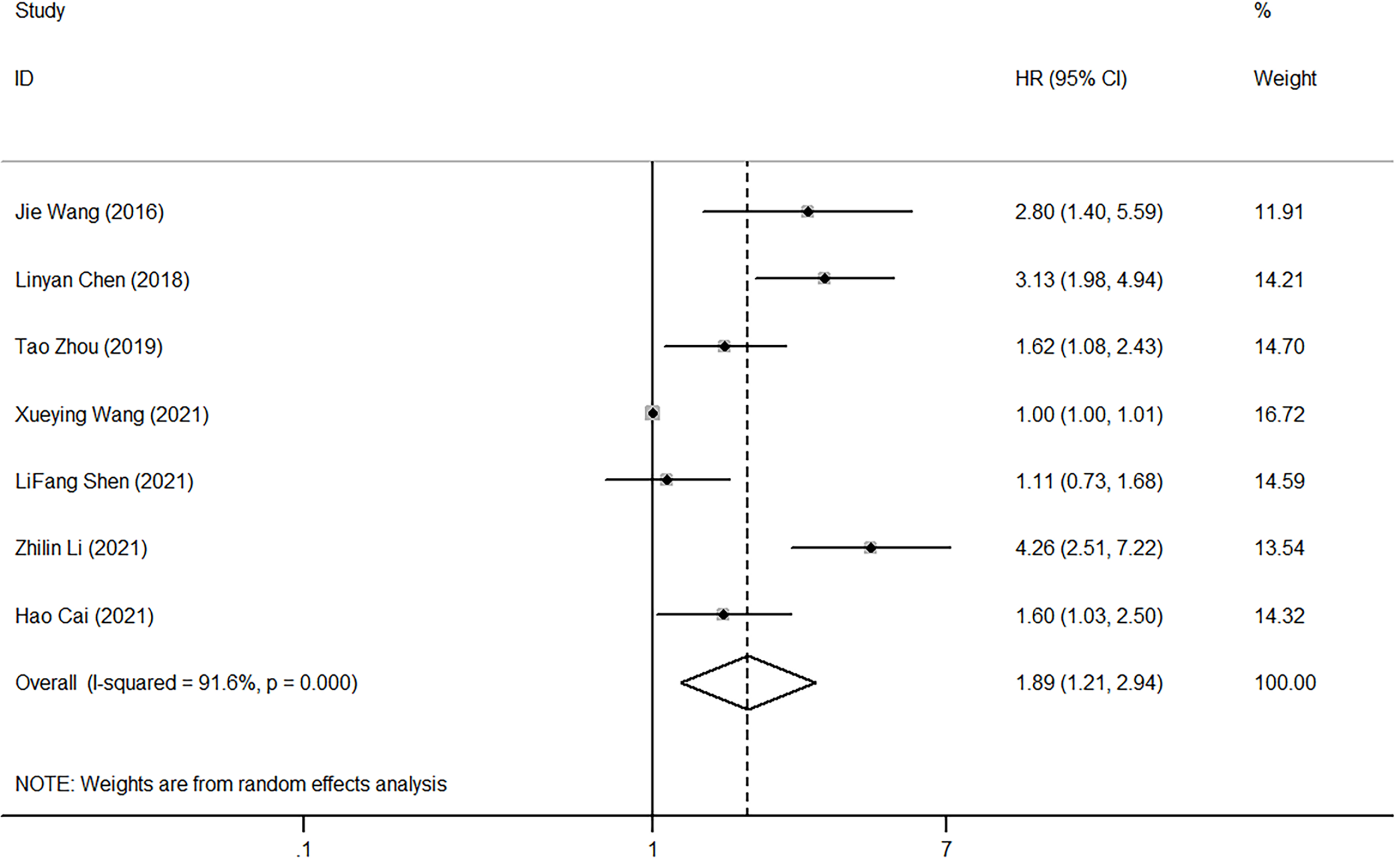
Forest plot of HR for the impact of PLR on OS. PLR, platelet-to-lymphocyte ratio; OS, overall survival; HR, hazard ratio; CI, confidence interval.

Only 8 of the 20 studies explored the relationship between PLR and OS. The random-effect model was used to analyze the effect of PLR on OS due to the presence of high heterogeneity (I2 = 91.6%, p<0.001). The pooled HR equaled 1.89 (95%CI, 1.21-2.94, p<0.001), indicating that high PLR could suggest poor OS (**Figure 6**).

A subgroup analysis was performed according to the diagnostic method, NOS score, sample size, and statistical model (univariate or multivariate). As shown in **Table 3**, the above diagnostic methods (I2 = 91.60%, p<0.001), NOS scores (I2 = 91.60%, p<0.001), sample size (I2 = 91.60%, p<0.001), and statistical model (univariate or multivariate) (I2 = 91.60%, p<0.001) all contributed to the overall heterogeneity. Subgroup analysis by NOS score suggested that high PLR predicted poor OS when NOS score was 7 (HR=1.62, 95%CI, 1.08-2.43) or 8 (HR=2.177, 95%CI, 1.198-3.955), but not when NOS score was 6 (HR=1.59, 95%CI, 0.5-4.29). Given that there were only two studies with a NOS value of 6, this finding needs further validation. Moreover, high PLR was related to poor OS for sample sizes less than 200 (HR=2.289, 95%CI, 1.428-3.667), while this result could not be obtained when sample sizes were more than 200 (HR=1.472, 95%CI, 0.790-2.741) (**Table 3**).

**Table 3:** Main results of the subgroup analyses for the impact of PLR on OS.

Analysis	Number	Fixed-effects model		Random-effects model		Heterogeneity	
		HR(95%CI)	P	HR(95%CI)	P	I2	Ph
Diagnostic method	7	1.003(1.000-1.006)	0.038	1.887(1.209-2.944)	0.005	91.60%	0.000
pathology	5	1.003(1.000-1.006)	0.043	1.758(1.054-2.931)	0.031	90.70%	0.000
others	2	2.220 1.614-3.055)	0.000	2.235(1.159-4.308)	0.016	76.30%	0.040
NOS	7	1.003(1.000-1.006)	0.038	1.887(1.209-2.944)	0.005	91.60%	0.000
6	2	1.003(1.000-1.006)	0.048	1.578(0.581-4.286)	0.371	88.20%	0.040
7	1	1.621(1.083-2.427)	0.019	1.621(1.083-2.427)	0.019		
8	4	2.033(1.618-2.554)	0.000	2.177(1.198-3.955)	0.011	85.20%	0.000
Sample size	7	1.003(1.000-1.006)	0.038	1.887(1.209-2.944)		91.60%	0.000
<200	4	2.124(1.665-2.709)	0.000	2.289(1.428-3.667)	0.001	71.60%	0.014
≥200	3	1.003(1.000-1.006)	0.046	1.472(0.790-2.741)	0.223	91.70%	0.000
Statistical model	7	1.003(1.000-1.006)	0.038	1.887(1.209-2.944)	0.005	91.60%	0.000
M	5	1.003(1.000-1.006)	0.043	1.758(1.054-2.931)	0.031	90.70%	0.000
U	2	2.220(1.614-3.055)	0.000	2.235(1.159-4.308)	0.016	76.30%	0.040

M, multivariate; U, univariate; HR, hazard ratio; CI, confidence interval.

### Publication bias and sensitivity analyses

**Figure 7:**
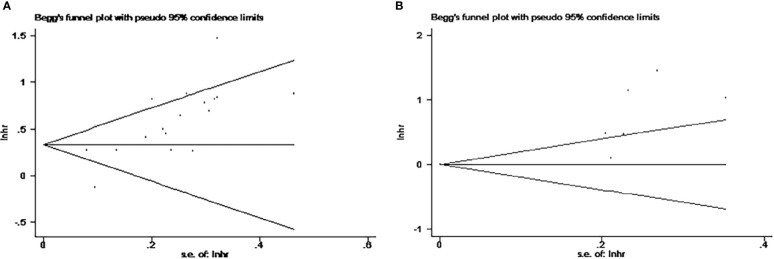
Begg funnel plots for publication bias. **(A)** Studies for the impact of NLR on OS. **(B)** Studies for the impact of PLR on OS. NLR, neutrophil-to-lymphocyte ratio; PLR, platelet-to-lymphocyte ratio; OS, overall survival.

Publication bias was assessed using Egger’s test and Begg’s test (**Figure 7**). When analyzing the impact of NLR on OS, publication bias was found in both testing methods (Egger’s test, p<0.001<0.05; Begg’s test, p<0.0016<0.05) (**Figure 7A**). Analyzing the impact of high NLR on OS revealed no publication bias by Begg’s test (p=0.072>0.05), but showed publication bias through Egger’s test (p=0.01<0.05). Due to limitations in the number of studies, NLR publication bias for PFS and DFS was not performed (**Figure 7B**).

**Figure 8:**
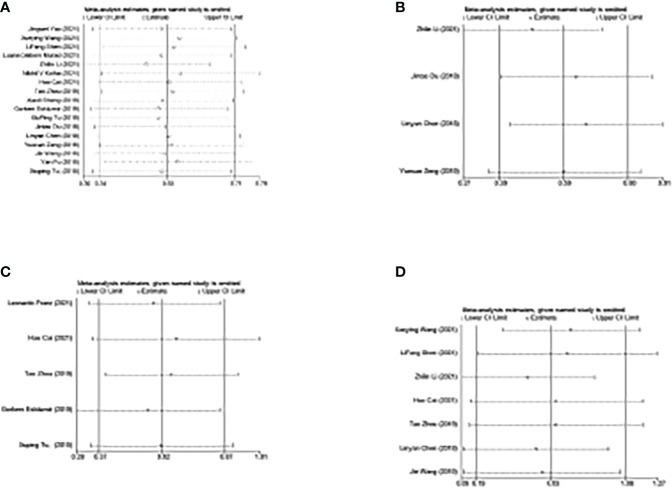
Sensitivity analysis of the corresponding included studies in each study. **(A)** Study for the impact of NLR on OS. **(B)** Study for the impact of NLR on PFS. **(C)** Study for the impact of NLR on DFS. **(D)** Study for the impact of PLR on OS. NLR, neutrophil-to-lymphocyte ratio; PLR, platelet-to-lymphocyte ratio; OS, overall survival; PFS, progression-free survival; DFS, disease-free survival.

Sensitivity analysis assessed the impact of individual studies on the overall results. As shown in **Figure 8**, the omission of one study did not affect the outcomes, indicating the stability and reliability of our results.

## Discussion

Biomarkers play an important role in the diagnosis of many diseases (43, 44), and urine biomarker monitoring has become an effective approach for molecular diagnosis and individualized drug use of bladder (45). At the same time, inflammatory response plays a vital role in the development, progression, and metastasis of cancer. And it is closely associated with death and recurrence in patients with malignancy after surgery (11, 46). Previous studies have shown that various validated indicators such as C-reactive protein (47) and interleukin-6 (48) are closely associated with the prognosis of cancers. Na Liu et al. concluded that both high NLR and PLR predicted poor prognosis in non-small cell lung cancer (49). A study by Song et al. on osteosarcoma also revealed that high NLR/PLR was associated with poor prognosis (50). And Yan Jiang et al. found the same predictive effect of NLR/PLR for esophageal cancer (51).

This study is the most comprehensive and complete meta-analysis of the predictive value of NLR and PLR in laryngeal cancer as is known to us. We included 5716 patients from 20 studies and confirmed that high NLR and PLR predicted poor OS. We also concluded that high NLR was associated with poor PFS and DFS. A recent meta-analysis published by Lin Yang et al. about the role of NLR in predicting the prognosis of head and neck squamous cell carcinoma is available (7). Compared with them, we narrowed the disease to laryngeal cancer, which is more in line with precision medicine. We also added PLR as a predictive index, making the assessment more comprehensive. In addition, Fangyu Yang et al. also conducted a meta-analysis of the relationship between NLR and laryngeal cancer (52), but we included more studies (20vs12 studies in Fangyu Yang’s) and more patients (5716vs3746 patients in Fangyu Yang’s). More studies and patients make our research more accurate and scientific. And we comprehensively analyze the impact of NLR and PLR on OS, DFS, PFS, which makes our conclusion of more clinical value. Notably, the similar results of the above two meta-analyses further support the results obtained from our study, giving more conviction to this study.

Numerous studies reported the relationship between NLR/PLR and the prognosis of laryngeal cancer, while their results are contradictory (22–26). Some studies confirm that high NLR/PLR predicts poor prognosis (22–24), but others fail to do so (22, 25, 26). Therefore, in this study, we performed a meta-analysis to accurately predict the predictive value of pre-treatment NLR/PLR in laryngeal cancer.

Our results show that high pre-treatment NLR predicted poor OS (HR=1.70, 95%CI, 1.41-2.04, p<0.001), PFS (HR=1.81, 95%CI, 1.47-2.23, p<0.001) and DFS (HR=1.86, 95%CI, 1.45-2.38, p<0.001). Similarly, high PLR was associated with poor OS (HR=1.89, 95%CI, 1.21-2.94, p<0.001). Subgroup analysis revealed that various factors such as diagnostic method, follow-up time, tumor type, study area, NOS score, cut-off values, and sample size were sources of heterogeneity. More studies and analyses are needed to explore the effects of subgroups. The reliability of our findings was verified by sensitivity analysis, but the tests showed a significant publication bias. Results of studies with significance are more likely to be reported and published than those with insignificance and invalidity (53). Based on this result, we judged that pre-treatment NLR and PLR might be vital indicators to predict the prognosis of laryngeal cancer. NLR and PLR are inexpensive and readily available blood-borne parameters, so we suggested hospitals paying attention to NLR and PLR values and providing appropriate treatment based on them.

Although many studies have reported the predictive role of PLR/NLR on cancer prognosis, the mechanisms have not been fully clarified (16). The increase of NLR and PLR levels suggests a relative decrease of lymphocytes and a relative increase of neutrophils and platelets. Two reasons for high NLR predicting poor cancer prognosis are available. One explanation is that, as a typical inflammatory cell, neutrophils represent the inflammatory state of the tumor (54–56). Neutrophils can produce vascular endothelial growth factors, hepatocyte growth factors, chemokines, intercellular adhesion molecules-1, IL-6, IL-8, transforming growth factor-β, and matrix metalloproteinases. All these secretions have been shown to promote angiogenesis, and cell growth, thus facilitating tumorigenesis and development (57–63). Evidence suggested that neutrophils could promote tumor metastasis and invasion (14, 64–66). Meanwhile, neutrophils can secrete reactive oxygen species, nitric oxide, and arginase to suppress lymphocytes and natural killer cells (67, 68). They can also inhibit T-cell proliferation through hydrogen peroxide and integrin Mac-1 (69). Therefore, high neutrophils indicate a poor prognosis of cancer. Another explanation is that lymphocytes, a fundamental component of the immune system, can reflect anti-tumor immunity (29, 70). Enough studies showed that lymphocytes could suppress the proliferation and metastasis of tumor cells and avoid the rise and spread (15, 55, 56). Therefore, a decrease in lymphocytes often indicates a poor prognosis for cancer. For PLR, the same two factors come into play. Besides the lymphocytes mentioned above, elevated platelets are also essential. Tumor cells can secrete inflammatory mediators (71), thrombopoietin, and leukemia inhibitory factor (72), which promote the differentiation of megakaryocytes to platelets (73). These secretions can also facilitate the activation of platelets (74, 75). The elevated platelets in turn release inflammatory mediators and vascular endothelial growth factor to promote tumor angiogenesis (16). Various studies reported that platelets could promote tumor growth and metastasis and fight immune responses (71, 76–78). Myriam Labelle’s study indicated that platelets induce tumor cells epithelial-mesenchymal transition through activation of TGF-β/Smad and NF-κB pathways, thereby promoting their metastasis (79). Thus, increased platelets are closely associated with tumor progression.

In short, NLR and PLR reflect the balance of tumor inflammatory response and anti-tumor immune response. Their increase indicates an inadequate immune response to tumors and the formation of an immune microenvironment conducive to tumor growth, thus enabling to prediction of the prognosis. In addition, various studies have shown that NLR is more effective in predicting cancer prognosis than PLR (39, 80). All of these coincide with the results of our study.

However, besides publication bias, our meta-analysis also had some limitations. First, significant heterogeneity existed in the HR for OS (NLR, I2 = 72.2%, p<0.001; PLR, I2 = 91.6%, p<0.001). Despite using sensitivity and subgroup analyses, it is not easy to accurately trace the source of heterogeneity. Second, all included studies were retrospective. Therefore, some prospective randomized studies were necessary to confirm our results. Third, when analyzing the impact of NLR on PFS/DFS, there are fewer eligible papers. The same applied when analyzing the relationship between PLR and OS. And we limited the language of publication to English so that we might have missed a lot of high-quality studies.

## Conclusion

Our results indicated that high NLR and PLR before treatment suggested poor prognosis in patients with laryngeal cancer. As inexpensive and easily accessible markers, NLR and PLR have the potential to become indicators to guide clinical treatment. In the future, further confirmation of our findings through numerous prospective studies is needed.

## Author contributions

XH wrote the manuscript with contributions from all authors. TT collected data. QS performed the analysis. WJ conceived and designed the study. All authors critically revised and approved the manuscript.

## Funding

This work was supported by National Natural Science Foundation of China Grant 81725004.

## Acknowledgments

We thank all the authors of the included studies.

## Conflict of interest

The authors declare that the research was conducted in the absence of any commercial or financial relationships that could be construed as a potential conflict of interest.

## Publisher’s note

All claims expressed in this article are solely those of the authors and do not necessarily represent those of their affiliated organizations, or those of the publisher, the editors and the reviewers. Any product that may be evaluated in this article, or claim that may be made by its manufacturer, is not guaranteed or endorsed by the publisher.
